# Harvard Odontological Society

**Published:** 1893-08

**Authors:** 

**Affiliations:** Harvard Odontological Society


### HARVARD ODONTOLOGICAL SOCIETY.

The monthly meeting was held at Young's Hotel, Boston, on March 30, 1893, at 6 P.M., President Eddy in the chair.

The paper of the evening was read by E. C. Briggs, M.D., D.M.D. Subject, "The Dentist as a Prescriber of Drugs."

(For Dr. Briggs paper, see page 567.)

### DISCUSSION.

*Dr. Keep.*—I would like to ask Dr. Briggs if, even in the matter of food-supply, there is not the same objection which he urged against the giving of drugs,—that you would not be able to see your patient frequently enough to observe its effects? Would it not be better even to leave that to the family physician, if necessary giving him hints as to what is needed? Would you be able to follow it up so well as the family physician, who is more familiar with the family history and with the patient?

*Dr. Briggs.*—My ground as regards food-values is, that you see the children in comparative health,—that is to say, in your relation as dentist a child is brought to you who may need, and you see he requires (provided you have been posted on food-values), a marked change or regulation of his diet, when ordinarily the physician has never been called. The child is not sick, and it is not a case where there is any drugging required; all it needs is simply a change of the diet. Now, if a child, through a false idea of its parents, has been fed on oatmeal every morning, and is showing symptoms of acid dyspepsia, and yet not ill enough to call in the family physician, you would simply recommend them to give it to the child every alternate morning, or only once a week, or stop giving it altogether, and in such a case you are not doing anything from which the patient might be in danger, as you would if you prescribed a cathartic. In prescribing a drug one should know the result. That person may be susceptible to the drug, and if you were a physician you would visit the next day and see how your patient was affected; but if you are a dentist, you do not go. That is the point where I contrast those two: drugs are usually prescribed for those whose condition is in some way below normal, whereas in hygiene and diet, in food-values, you enter upon their use in what is ordinarily called health.

*Dr. Keep.*—Have you observed many cases in which much change has been brought about by a change of diet?

*Dr. Briggs.*—In regard to what?

*Dr. Keep.*—In regard to conditions of the mouth and development of the teeth.

*Dr. Briggs.*—I can't say so much about the teeth, because I think that is the hardest thing to get at. I have observed changes in condition brought about by a change of diet which I felt were bearing on the mouth,—notably in abscess in the mouth, dyspeptic troubles, and all those things which more or less affect the teeth.

*Dr. Clapp.*—I suppose there are few questions that are asked us oftener than in regard to diet. I have one form of reply to these interrogations; it is this: Feed your children so that the entire system shall be brought up to the highest development. I do not believe in any specific dieting,—that is to say, I do not believe in feeding phosphates to make teeth, for this reason: in the mixed, ordinary, plain food there is sufficient material for the building up of the different parts of the system. Now, what is the use of overloading the system with phosphates when the conditions are such that the phosphates will not be assimilated? So I make this rule:

advise that the feeding shall be simple, plain, and nourishing, without specific anticipation of improving the teeth by giving phosphates or things of that kind. I have one special case in mind,—a little patient who is now, I think, nine or ten years of age. When this little girl was born she was perfectly healthy and was, and is, an ordinarily healthy child. She was fed on rigid principles of diet, I think, until she was five or six years of age,—probably she had no meat at all, but she had the various kinds of grains that are on the market called "health foods," and plenty of milk and fruit. The mother expected that the teeth, especially the sixth-year molars, would be the ideal of perfection. The result is that they are about as poor sixth-year molars as I have ever seen. They are of the kind that you might designate as "honeycombed." They are filled with little pin-hole cavities, the seams running in the enamel from the centre outward are badly decayed. When they first began to decay I filled them with cement, cutting them out as much as appeared to be necessary, but at each subsequent sitting I found that the decay had gone still farther and that the smallest pits had become quite large cavities. Now, I do not know whether this was a proper case of feeding or not, and I don't know but that the teeth might have been very much worse if the child had been fed in any other way, but it is a case where this kind of feeding did not make good teeth.

*Dr. Smith.*—I think feeding has become quite a science. The discussion has brought to my mind a clipping which I took from the Boston *Medical and Surgical Journal* some years ago. I have it in my scrap-book. That article contained a quotation from a German medical periodical, giving the experience of some German scientists experimenting on guinea-pigs with the different kinds of food-supplies, and, if I remember rightly, giving them the phosphates in the form of syrups for their bony structure. An examination in all those cases proved that the phosphates were passed in the fæces.

I have known cases where in a family of children some of them having poor teeth were given phosphates, all of them living on the same diet,—that is, as near as a number of children at the same table would be apt to, some of them eating one thing and some another,—and yet the children having poor teeth were none the better for the administration of the phosphates; and these things teach us that while we may see teeth that need the phosphates, still there is no direct route that will take them into the system and carry them to the teeth. Even if there was such a route, and you could depend on perfect assimilation, I believe there would still be something needed for the preservation of the teeth and making them harder. I believe the teeth need exercise. Take an athlete. He is particular about his diet, yet if his arm be strapped down by his side the greatest possible care in dieting will not harden the muscles of that arm. He must use those muscles constantly; and not only must he exercise, but produce friction with towels and rub-downs to bring the blood there and give the parts new vigor. Now, when we see teeth of poor quality the circulation is generally at the low ebb, so to speak, and they need exercise; much harder foods should be used for the purpose,—not substances which require little or no mastication,—and this should be followed by a thorough rubbing of the gums and teeth with proper brushes. This has a great deal to do with the benefits derived from food-supplies, and is a great factor in the attempt at improvement of the quality of the teeth, and particular stress should be laid upon it, I think, in advising patients in the care of their teeth.

*Dr. Werner.*—I agree heartily with what Dr. Smith has said. I think the problem of elimination of effete substances needs study more than that of feeding. We must work; we must produce a hunger in the tissues, in the molecules; then assimilation will take care of itself. Preventive medicine stands highest, and the days of wholesale and indiscriminate prescribing of drugs are gone by. In the daily struggle for a living, for an education, we neglect the laws that govern health, and physical culture is little thought of when most needed. Gymnasiums were not as popular twenty years ago as they are to-day. We cannot in the course of four or five years build up a degenerated dental organization. It will take several generations to do that.

*Dr. Taylor.*—I would like to ask Dr. Briggs if he has noticed any change in the conditions about the gums in pyorrhœa which he thought was due to any dietetic change? I think he must have had something in view regarding that when he wrote, and I would like to know if there is anything that will have any good effect on that pathological condition.

*Dr. Briggs.*—I shall have to answer that at present in the negative; it is only a theory with me and I have nothing yet to present. Some day perhaps I shall have,—at least I hope to. I cannot help feeling that that is the direction in which we will find relief.

In speaking of diet, you will remember in my paper I said "Hygiene and Diet." You cannot do anything with the latter unless you have the former. You might as well expect to produce offspring from the female without bringing the male. You must remember that the two go together. In order to be utilized, foods have to be oxidized, and in proportion as they are oxidized properly are they used in the system. In the matter of supplying phosphates, it may be that we have not as yet found the proper way to introduce them in order that they may be assimilated. We make the general statement that the ordinary food contains the proper amount of phosphates, but at the same time it is a fact that we meet with many instances where the conditions are such that the phosphates are not assimilated, and unless those conditions are altered the tendency is not to utilize the phosphates, but to eliminate them. We give a child a certain amount of starchy food, and in eating that with the proteids, the oxygen that is taken oxidizes the starchy food first, then if there is not enough to oxidize the proteids, they are cast off, and you will find the phosphates in the fæces. It might serve as an illustration to compare this action with feeding a couple of pigs, a big one and a little one. If there is no more than the big one can take, he will get it all, and so if the starchy foods are greatly in excess of the proteids and only a certain amount of oxygen is given to that child, either from lack of proper exercise or from lack of good hygienic surroundings, why, then the starchy foods will grab the oxygen and the phosphates will not be taken into the system. In bony diseases similar results are produced by this perverted nutrition; for instance, the disease called rickets belongs to improper diet and improper hygiene, —correct these and you correct the rickets.

*Dr. Smith.*—I would like to ask the essayist if he believes that it is possible, or if it is not possible, if it is wise, to make the attempt to discover some drug or drugs that may be administered in the incipient stages of an alveolar abscess to abort it? To-day we treat alveolar abscesses entirely surgically, that is to say, the majority of dentists do. It is not necessary for me to go through the treatment that we use. The remedies that we use are topical, and in most cases we do not enter upon any systemic treatment. I do not know that it is necessary at all to come to the defence of Dr. Taft, but the essayist speaks of his paper and draws the conclusion that the field into which he has entered is rather a dangerous one for the practitioner of dentistry. If I have drawn the proper conclusion from the essay presented this evening, it is the claim of the essayist that the administration of drugs should be left wholly to the physician. As I recall Dr. Taft's paper, I believe he went into the administration of drugs to a limited extent, notably in cases of alveolar abscess, and he cited those cases which were benefited by his treatment. Now, the drugs which he claimed would benefit or cure these lesions were supported by tabulated records of his cases. But he was honest enough to say in his paper that the efficacy of any method could not be established until many cases had been tabulated, yet in homœopathy they claim that they do cure alveolar abscesses by the administration of homœopathic drugs. They may differ as to the attenuation of medicine to be given,—some using the high potencies and others the low,—yet they all agree that they aid very much and sometimes cure alveolar abscess. Now, in my own practice I have witnessed cases where patients, physicians in homœopathy, have taken their remedies for alveolar abscess during the time I was treating the abscess surgically, and they seemed to get along unusually well. It may have been simply a coincidence; I won't jump at conclusions, as many of our homœopathic friends are apt to do. And, by the way, that reminds me of an incident which is a little foreign to the subject, but so patent an illustration that I can't help speaking of it. The wife of a prominent homœopathic physician went to a dentist to have her teeth cleaned. The teeth were in a very advanced stage of pyorrhœa and one of them was very loose; after removing the tartar this tooth seemed to be looser; thereupon the physician claimed that the cleaning of teeth was injurious, and the teeth should never be cleaned. He is a gentleman who stands very high in homœopathy, but if he arrives at all his conclusions in that way, his science is considerable of a farce.

Now, the school of allopathic physicians claim they can do nothing in the way of systemic treatment for alveolar abscess; that the matter is on the surface, and should be treated there. If that is an established fact, then there is nothing we can do but to let it go on through the swelling and regular abscess, but if there be another way to treat alveolar abscesses and to abort them, then we are very unwise not to look into it. This is what Dr. Taft is doing, and to that extent I wish to encourage him in his investigation and in his administration of drugs.

*President Eddy.*—I would like to ask Dr. Keep, as a graduate of medicine, if the old schools do not give sulphide of calcium to prevent an abscess forming, with good results?

*Dr. Keep.*—I don't know whether that is the practice or not. I know it has been the general rule to give cathartics when there was any local inflammation.

*Dr. Smith.*—I would like to have Dr. Briggs answer my question. I talked a good while after asking it, and don't blame him for forgetting it.

*Dr. Briggs.*—My reply to that question would be that there is no doubt that in cases of alveolar abscess, or in any like trouble, there are drugs that can be given to palliate or alleviate the symptoms and make the case milder than it would be without treatment, but as for aborting it, I do not understand that any drug can be given to do that. The condition of alveolar abscess is a different thing from the condition of a patient who has boils. Boils are the result of a poor condition of the blood, whereas if a man has an abscess, as a rule, there is a local cause, and if that is once started you cannot control it by giving drugs to be taken into the general system.

To illustrate my point, a patient came to me with an alveolar abscess forming. The tooth was very tender, and its history indicated that it had been filled in the roots. If I had known beforehand that the patient was going to have an abscess, I could have tried refilling it, thinking perhaps it was not filled as thoroughly as it could have been, but it was too late to do that, the abscess was formed and the patient was in a very nervous condition. I gave her topical remedies to be applied, and told her to go home and call in her physician. Now, a dentist cannot properly attend to a case of that kind. If he gives her systemic remedies, he has to go and see her every day as a physician does, and that, of course, he cannot do and attend to his office practice,—something has to be sacrificed. She certainly ought to stay in the house, and not go to his office, and if he is practising a specialty he will hardly find time to run out and see how that pill worked he gave last night.

Perhaps some of the members of the Society have noted a fact that has occurred to me from questioning patients who have pyorrhœa, and that is, that in a great majority of cases—and I cannot say that I ever saw a single exception—there has been a rheumatic or gouty diathesis in the family. I have in mind three cases which were as severe as I ever saw, and in each instance there was a marked rheumatic ancestry.

*Dr. Clapp.*—I saw two or three weeks ago a model of a mouth where the lower teeth were very much out of place. They seem to have been thrown upward and forward, so that the arch was very much enlarged and extended over the superior arch in some parts of the mouth. There was a very wide separation between the incisors, and it would seem that some influence had been at work to throw the teeth upward and outward, and it was said that rheumatism was the cause of this change in the mouth. The patient had been a great sufferer from rheumatism, the joints being almost anchylosed. I have held for years that many of the ills suffered by the teeth arise from this disease.

*Dr. Briggs.*—If you admit that, there comes in your hygiene and diet.

*Dr. Werner.*—I wish to speak of a case that is a good illustration of our inability to abort alveolar abscess. While operating for a gentleman in a common cavity of decay, last spring, I found the pulp dead. I cleaned out the canal, treated it several times, and filled it temporarily with gutta-percha. Two months after, the patient came to my office with a swollen cheek. The tooth was but slightly sore and could be operated upon very comfortably. I took out the temporary filling, made aseptic applications, and dressed it. Instead of getting better, it gradually became worse, and at the end of two weeks there was so much inflammation that a surgeon was called in, who injected cocaine, made an incision on the inside, and four days afterwards another incision on the outside. It seemed as if there might be some obstruction in Steno's duct which was almost as large as a pencil. Poultices were worn, and I applied, or attempted to apply, two leeches, but they would not take hold. This is a severe case, and I feel that dentists ought to know whether it should be treated systemically. The patient was very sick, and whether the abscess in the cheek was caused by that second molar is an interesting question. I think it was.

*Dr. Briggs.*—What proof have you of that?

*Dr. Werner.*—That was the only tooth that was sore, and yet there was no absolute proof, as the root was perfectly clean; I could detect the odor of the campho-phenique with which I treated it last spring.

*Dr. Grant.*—Perhaps there was an abscess coexistent with the one you speak of.

*Dr. Page.*—Did you notice the occlusion?

*Dr. Werner.*—The occlusion was normal. In the sorest condition it did not interfere with his bite.

*Dr. Smith.*—Why not extract the tooth?

*Dr. Werner.*—Evidently the patient was developing an abscess. He would in that case have suffered and lost his tooth.

*Dr. Smith.*—I should expect that the root of that tooth penetrated the antrum, and I should have gone up there to see if I could not find the cause of the trouble.

*Dr. Werner.*—The inflammation was over the malar bone and was deep-seated. That is where the pus formed.

*Dr. Shepherd.*—Was there any soreness under the eye?

*Dr. Werner.*—The whole side of the face was very much swollen, including the eye.

*Dr. Blaisdell.*—While on the subject of abscesses I wish to report a case from the Dental School the past winter. A young lady had the pulps removed from the right superior bicuspids and the canals filled in the usual way. Shortly after, she returned with an abscess over these bicuspids. The canals were opened and the teeth treated through the canals for two months with no benefit. Every time the dressing was put in, even though only twenty-four hours elapsed, she returned with great inflammation, until the patient was discouraged and wished to have them removed. It was suggested that the student should fill the roots and cavities permanently and then open from the outside directly through the alveolar wall, and with a burr remove what necrosed tissue was there. He did so, and found a large cavity that ran forward from the first bicuspid almost to the central incisors and backward to the second molar. He gave it a thorough burring, washing with peroxide of hydrogen. In two weeks the patient returned with everything healed and no signs whatever of having had an abscess. That was a case that was about to be given up, but a thorough surgical operation brought about a cure.

*Dr. Briggs.*—I think this only points out the moral of my paper. We want more surgical knowledge, and I should say in Dr. Werner's case either he wanted a little more surgery, proving whether or not the tooth was the offending member, or else the general surgeon should have been referred to earlier. We should not take it upon ourselves to treat an abscess because it is adjacent to teeth any more than the oculist who, in his examination, comes upon a middle-ear disease would attempt the treatment of that. He would send it to the aurist.

I had a case which was similar to the one which Dr. Blaisdell speaks of. The patient came from out of town, and had had a great deal of trouble with an abscess over a bicuspid. She said the pus discharged so freely that she soaked three handkerchiefs in a night. Her dentist had tried in vain to heal it and had advised the extraction of the tooth, but she did not wish it removed. I placed the case first in my brother's hands to have the root carefully filled, and I then made an examination. At first I thought it was a diseased antrum. There was a discharge into the left nostril, and in the post-nasal examination I could see the pus welling through the left nasal fossa. I began the treatment with opening into the antrum, and found it clean and dry. I then made incisions and introduced the burr over the bicuspid, cutting out quite a piece of the alveolus that was dead. After treating the wound with antiseptic sponge, as described in the *Independent Practitioner* some ten years ago, the patient was discharged cured. That was a surgical operation entirely, and I think we can do a great deal with surgery. We have the field and I think we had better stick to it, and we will find how much more good we will be able to accomplish in that line.

*Dr. Keep.*—I think we ought to be very careful about making positive statements regarding the value of drugs for aborting abscesses. A large number of cases which at the outset seem to be very threatening and are apparently developing into abscesses disappear without any active treatment. It is a difficult thing to say surely that an abscess will develop,—it may, and it may not; and in view of this fact it is well not to be too sure that favorable issues are the result of any general treatment we may have adopted.

HENRY L. UPHAM, D.M.D.,
*Editor Harvard Odontological Society.*

